# Analysis of Genetic Alterations in Ocular Adnexal Mucosa-Associated Lymphoid Tissue Lymphoma With Whole-Exome Sequencing

**DOI:** 10.3389/fonc.2022.817635

**Published:** 2022-03-11

**Authors:** Andi Zhao, Fangtian Wu, Yue Wang, Jianyong Li, Wei Xu, Hu Liu

**Affiliations:** ^1^ Department of Ophthalmology, The First Affiliated Hospital with Nanjing Medical University, Nanjing, China; ^2^ The First Clinical Medical College, Nanjing Medical University, Nanjing, China; ^3^ Department of Hematology, The First Affiliated Hospital of Nanjing Medical University Jiangsu Province Hospital, Nanjing, China; ^4^ Key Laboratory of Hematology, Nanjing Medical University, Nanjing, China; ^5^ Collaborative Innovation Center for Cancer Personalized Medicine, Nanjing Medical University, Nanjing, China; ^6^ Department of Medical Oncology, Fudan University Shanghai Cancer Center, Shanghai, China; ^7^ Department of Oncology, Shanghai Medical College of Fudan University, Shanghai, China

**Keywords:** ocular adnexal lymphoma, MALT lymphomas, *IGLL5*, somatic mutation, pathogenesis

## Abstract

Next-generation sequencing studies on ocular adnexal marginal zone lymphoma of mucosa-associated lymphoid tissue (OAML) have to date revealed several targets of genetic aberrations. However, most of our current understanding of the pathogenesis and prognosis of OAML is primarily based on studies conducted in populations from Europe and the US. Furthermore, the majority were based on formalin-fixed paraffin-embedded (FFPE) tissue, which generally has poor integrity and creates many sequencing artifacts. To better investigate the coding genome landscapes of OAML, especially in the Chinese population, we performed whole-exome sequencing of 21 OAML cases with fresh frozen tumor tissue and matched peripheral blood samples. *IGLL5*, as a novel recurrently mutated gene, was found in 24% (5/21) of patients, with a higher relapse rate (P=0.032). In addition, mutations of *MSH6*, *DIS3*, *FAT1*, and *TMEM127* were found in 10% of cases. These novel somatic mutations indicate the existence of additional/alternative lymphomagenesis pathways in OAML. Moreover, the difference between our and previous studies suggests genetic heterogeneity of OAML between Asian and Western individuals.

## Introduction

Ocular adnexal lymphomas (OAL) are rare, accounting for 1-2% of non-Hodgkin’s lymphomas (NHL) and 7-8% of extranodal marginal zone lymphomas (1, 2). The most common subtype of the latter occurs in the mucosa-associated lymphatic tissue (MALT) and is known as ocular adnexal marginal zone lymphoma of mucosa-associated lymphoid tissue (OAML), presenting in orbital soft tissue, eyelids, conjunctiva, and lacrimal draining apparatus (3).

The molecular pathogenesis of OAML has not yet been fully understood. The current prevailing view is that MALT lymphoma arises from a background of chronic inflammation, i.e., microbial infection and autoimmune disorders, at various mucosal sites while acquired genetic changes accelerate the malignant transformation, such as by activating canonical and/or non-canonical nuclear factor-κB (NF-κB) pathways (4, 5). Interestingly, MALT lymphoma from different sites of the body is associated with distinct etiologic factors and genetic aberrations. Chlamydia spp. infection has been reported as a potential trigger for OAML in some geographical areas but not in Chinese institutions (6–11). In addition, recurrent chromosomal abnormalities of MALT lymphomas, including t(11;18) (*BIRC3*/*MALT1*), t(14;18)(*IgH*/*MALT1*), t(1;14)(*BCL10*/*IgH*) and t(3;14)(*FOXP1*/*IgH*), have rarely been found in OAML (12–15). These findings indicate that either infection or structural chromosomal abnormalities play only a minor role in the pathogenesis of OAML.

Recently, several next-generation sequencing (NGS) studies have uncovered the genetics of OAML in some detail. Constitutive activation of NF-κB signaling by mutation or copy number alteration is a hallmark of OAML, including frequent inactivation of *TNFAIP3* (~30%) and activating variants of *MYD88* (7%~25%) (4, 15, 16). Moreover, histone-modifying genes are frequently affected, including CREBBP (9%~25%), *KMT2D* (6%~22%), and *TBL1XR1* (6%~19%). Moreover, immune surveillance genes are mutated in ~10% of cases (12, 17).

However, all of these NGS studies were based on formalin-fixed paraffin-embedded FFPE samples, resulting in unreliable sequencing data due to DNA fragmentation and chemical modification and reducing their sensitivity, specificity, and accuracy (18–21). Moreover, to date, only two studies performing whole genome/exome sequencing (WGS/WES) have been published, both with minimal cases of OAML (13 and 10, respectively) (17, 22).

Thus, to further understand the genomic basis of OAML derived from Chinese patients, we applied WES in 21 OAML cases using fresh frozen (FF) specimens and paired peripheral blood. Furthermore, clinical data were analyzed to investigate the prognostic value of recurrent mutations in OAML.

## Materials and Methods

### Patients

Patient samples were obtained from material archived at The First Affiliated Hospital of Nanjing Medical University between 2013 and 2015. All samples were surgically resected before any anti-tumor treatment and then immediately frozen in liquid nitrogen and stored at -80°C. Partial tissues of these samples were immediately sent for clinical, histological diagnosis, and the remaining portion was excised from the resected neoplasm for DNA extraction. These patients were definitely diagnosed based on the laboratory tests following the World Health Organization 2008 classification (23). Hematoxylin and eosin (HE) and immunohistochemical staining were evaluated for lymphoma diagnosis, including CD20, CD79a, CD3, CD23, Cyclin D1, CD5, CD10, Bcl-2, Bcl-6 together with immunoglobulin kappa, lambda light chains, and Ki-67. For cases in which immunophenotype could not be determined, the diagnosis was complemented with genetic analysis to exclude lymphoproliferative disorders. Also, we evaluated the serum IgG4 and lactate dehydrogenase (LDH) to exclude IgG4-related orbital disease (24).

### DNA Exaction and Whole-Exome Sequencing

Twenty-one patients diagnosed with OAML were finally selected for further study. HE staining and immunohistochemical evaluation showed that the FF samples contained at least 70% lymphoma cells. Tissue DNA was purified from the remaining portion of the FF sample, while germline genomic DNA was from paired blood samples. According to the manufacturer’s guidelines, total blood and tissue DNA was extracted using the DNeasy Tissue and Blood kit (Qiagen, Valencia, CA). The DNA concentrations were then measured with NanoDrop 2000 ((ThermoFisher Scientific, Shimadzu, Japan). The DNA concentration isolated from these samples were ranged between 50 and 2800 ng/ul with A260/280 ratio between 1.8 and 2.0. Due to poor quality, three patients’ germline genomic DNA from blood samples was excluded. Thus, sufficient amounts of DNA purified from 21 tumors and 18 paired blood samples were selected for further study.

Genomic libraries were generated using the Illumina Paired-End Sample Prep Kit for whole-exome sequencing, enriched using the Agilent SureSelect Human All Exon 50Mb Kit (Agilent, CA, USA), and sequenced on an Illumina HiSeq platform using a PE150 bp paired-end protocol. The tumor and paired peripheral blood DNA from 21 OAML patients were subjected to whole-exome capture and sequencing, targeting a mean coverage of 63 times. On average, 99% of exonic bases were covered by at least 10 sequencing reads. This study was approved by the Institutional Review Board from The First Affiliated Hospital of Nanjing Medical University, and written informed consent was obtained from all subjects after full explanation.

### DNA Sequencing Analysis and Data Processing

FASTQ files comprising reads from whole-genome sequencing were first tested for quality using FastQC. Adaptor sequences and low-quality reads were trimmed using Trimmomatic. Alignment was then performed using the Burrows-Wheeler Aligner (BWA) to map reads to the human GRCh38 reference genome. PCR duplicates were marked using Picard. Somatic variant calling was performed using GATK4 with the default parameters. The germline mutations detected from 18 respective paired blood samples were used to generate a negative-control library for three cases without paired blood samples. The setting for filtering was as follows: 1) the Phred quality score was set at >33. 2) Known single nucleotide polymorphisms (SNP, db137). 3) Variant with a frequency > 0.0001 in the 1000 genomes project data (1000 Genomes Project Consortium 2015). The remaining variants were analyzed manually using Integrative Genomics Viewer (IGV) to exclude germline variants and unreliable sequencing errors. In addition, pathogenic mutations were rescued from filtered mutations according to the published literature (4, 13).

### Pathway Analysis

To compare the mutation loads between different pathways reported previously, all filtered genes were categorized according to these pathways with the aid of *KOBAS* (http://kobas.cbi.pku.edu.cn/kobas3). Results were combined pathway annotations from the Kyoto Encyclopedia of Genes and Genomes (KEGG), BioCarta, PID, PANTHER, and Reactome databases. Furthermore, to explore more novel pathways involved in the pathogenesis of OAML, we assigned genes that contained nonsynonymous mutations to gene sets and interrogated the canonical pathways using KEGG. When calculating the mutation rates of different pathways, the total number of mutations was divided by the coding regions of all genes belonging to that gene set.

### Statistical Analysis

The Kruskal-Wallis-H test was utilized to assess quantitative differences between subgroups and the Mann-Whitney-U test was used to compare mutational burden between samples with and without somatic mutations (15). Correlations were estimated by applying the Spearman rank correlation test. Fisher’s exact or the chi-square test was used to compare qualitative data, including clinic characteristics with or without *IGLL5* mutations and distinct mutant frequencies from various regions. All statistical calculations were performed using SPSS version 24 or R (3.1.0) statistical package. A statistical significance threshold of *P*< 0.05 was assumed in all analyses.

## Results

### Clinical Features and Somatic Mutational Landscape of OAML Patients

In this study, we selected 21 OAML cases according to immunohistochemical evaluation. The available primary clinical and pathologic characteristics were summarized in **Table 1** and **Supplemental Table 1**. All patients had undergone surgical excision for tumor lesions. After surgery, the first-line treatment included a watch and wait strategy for seven patients (33%), local radiotherapy for seven patients (33%), systemic chemotherapy for four patients (19%), and a combination of radiation therapy and systemic chemotherapy for three stage IV patients (14%). During a median follow-up period of 70 months (range 18 to 84), four patients suffered a recurrence, including two with stage I disease, one with stage II disease, and another with stage IV disease. No patient died of disease, but one died of an unrelated cause, and 16 remained in remission. Notably, there was a significant mutational burden difference among patients with different stages (p=0.009). After pairwise comparison, we found that stage II patients harbored more somatic mutations than those in stage I (p=0.038), while no difference was seen between stages II and stage IV. No significant mutational burden difference was observed between sex or ages.

**Table 1:** Clinical and staging course of patients with ocular adnexal extranodal marginal zone B-cell lymphoma.

Characteristics	Total patients (n=21), (%)
**Age at diagnosis (years)**	
<55	7 (33%)
55-65	10 (48%)
>65	4 (19%)
**Sex**	
Male	12 (57%)
Female	9 (43%)
**Ann-Arbor Stage at diagnosis**	
I	16 (76%)
II	3 (14%)
IV	2 (10%)
**Localization at diagnosis**	
Orbita	15 (71%)
Conjunctiva	4 (19%)
Lacrimal gland	2 (10%)
**Laterality**	
Right	12 (57%)
Left	9 (43%)
**Treatment after surgery**	
Radiotherapy	7 (33%)
Immunochemotherapy	4 (19%)
Radiotherapy + Immunochemotherapy	3 (14%)
Watch and wait	7 (33%)
**Clinical outcome**	
Remission	16 (76%)
Relapse	4 (19%)
Dead of lymphoma ^1^	0 (0%)
**Proliferation index Ki-67, (%)**	
<10%	12 (57%)
10%-30%	9 (43%)
**Observation period (months)**	18-84 (mean:70)

^1^One died of an unrelated cause

**Figure 1:**
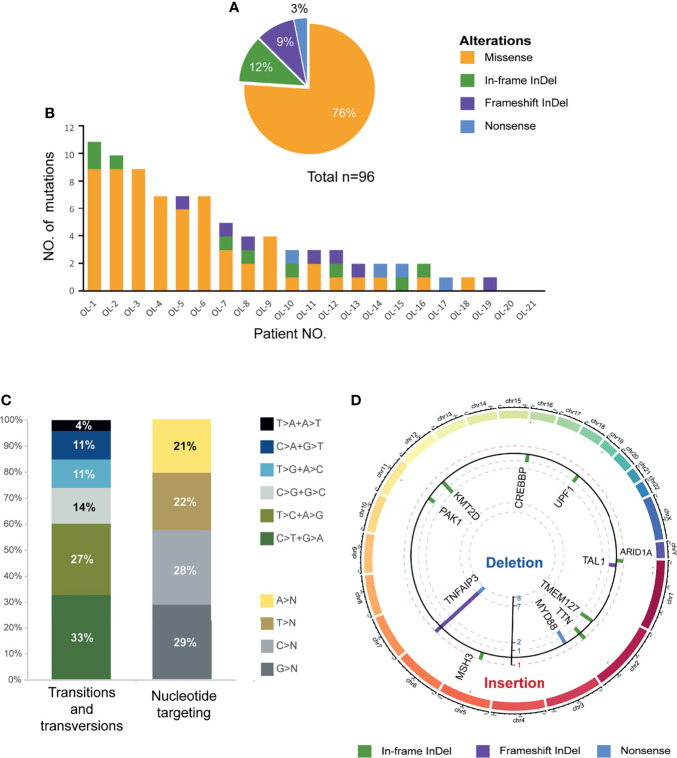
Overview of somatic mutational signature. **(A)** Pie charts showed the breakdown of different nonsynonymous mutation types in coding regions, including missense SNVs, frameshift insertions/deletions, in-frameshift insertions/deletions, and nonsense SNVs. **(B)** Number and type of nonsilent somatic mutations identified in the 21 discovery coding genomes. **(C)** The pattern of nucleotide substitutions in the discovery genomes revealed a predominance of transitions over transversions (47:29, ratio of 1.62) and a preferential targeting of G and C nucleotides (58% affecting G/C compared with 42% affecting A/T nucleotides). **(D)** Circo plot representing indels and nonsense mutations (n=23) mapped to different chromosomes scaled by the mutated sample counts (y-axis showed the unique samples count). The columns outside track represented the gene symbols with in-frameshift/frameshift insertions, while the columns inside track represented the in-frameshift/frameshift deletions or nonsense mutations.

To explore somatic mutations in the OAML coding genome, we performed WES on paired tumors and normal DNA from these patients. After filtering synonymous changes, known SNPs, sequencing artifacts, and germline variants, we identified 96 somatic, nonsynonymous variants involving 66 mutated genes in these cases, including 73 missense, 11 in-frame shift indel, 9 frameshift indel, and 3 nonsense mutations (**Figure 1A** and **Supplemental Table 2**). The mean number of mutations per tumor was 4.62 (range 0-11), observed in 19/21 (90%) cases (**Figure 1B**), consistent with previous findings (15, 17). The occurrence of variant allele frequencies ranged from 8% to 68%, reflecting homo- and heterozygous mutations. No sex or age difference was observed in the median number of mutations or single-nucleotide variants (SNV) patterns (data not shown).

The SNV spectrum analysis revealed an aging signature with the prominence of C>T substitutions at NpCpG trinucleotides, which was previously detected in other B-cell lymphoproliferative disorders (**Figure 1C**). Furthermore, **Figure 1D** showed the mutational spectrum of the indel and nonsense mutation in WES cohorts. Remarkably, *TNFAIP3* manifested the most frequent truncating mutations and deletions consistent with the loss-of-function of tumor suppressor, with 7 frameshift deletions, 1 frameshift insertion, and 1 nonsense mutation. On the contrary, no genes showed a similar enrichment of insertions in OAML.

### Identification of Recurrent Genetic Alterations in OAML by WES

**Figure 2:**
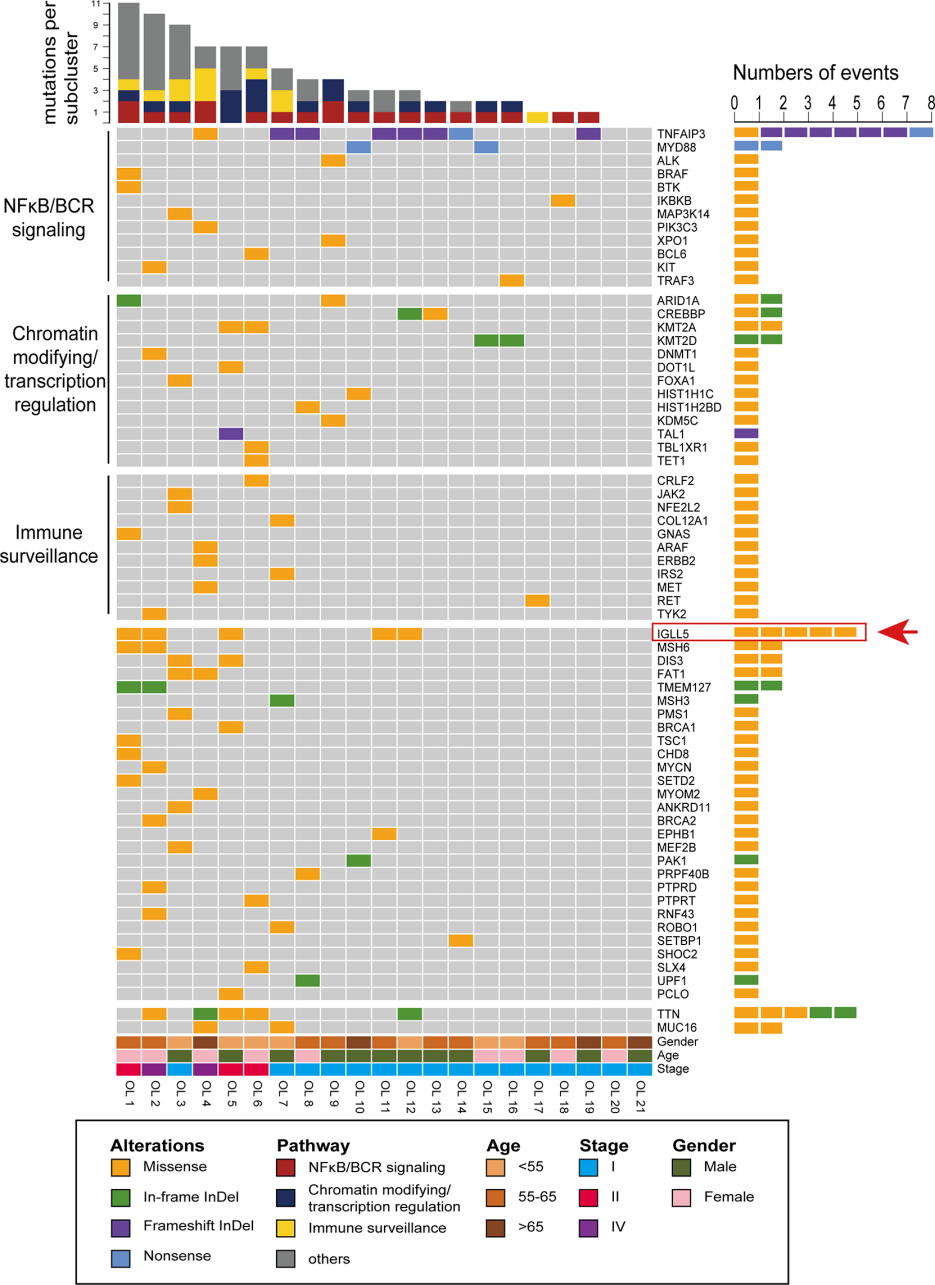
Recurrent targets of genetic alterations identified in OAML. The heatmap plot of the somatic mutations in the studied entities showing all nonsynonymous mutations detected by whole-exome sequencing (WES). Each column represents an ocular adnexal marginal zone B-cell lymphoma (OAML). Each row represents a gene divided by pathway and ordered from top to bottom in descending order of the tumor’s detection frequency. When multiple mutations occur within the same gene, the most harmful mutation is displayed.

As shown in **Figure 2**, a total of 13 genes were recurrently affected in more than one patient (≥2/21, 10%) with OAML. The most frequently mutated gene was *TNFAIP3* (8/21, 38%). Five others each occurred in 10% of cases reported previously, namely *MYD88*, *ARID1A*, *CREBBP*, *KMT2A*, and *KMT2D* (also known as *MLL2*. These genes point to the molecular deregulation of specific pathways in OAML, including NF-κB/BRC signaling (mutated in 81% cases), chromatin remodeling/transcriptional regulation (57%), and immune surveillance (29%).

Furthermore, several genes were revealed for the first time as mutation targets in OAML, affecting a relatively high proportion, including *IGLL5* (24%), *MSH6* (10%), *DIS3* (10%), *FAT1* (10%), and *TMEM127* (10%). *IGLL5* was the second most commonly-mutated gene, with 5 of 21 samples harboring mutations. Moreover, *IGLL5* is a surrogate light chain involved in B-cell development, which to our knowledge has not been reported in OAML to date. *MSH6* is a DNA mismatch repair (MMR) gene discovered in our cohort. The DNA MMR proteins increase replication fidelity and genome stability, and their defects reportedly play a carcinogenic role in different tumors (25, 26). Two alterations of *TMEM127* were both frameshift deletions (**Figures 1D**, **2**), which may be accompanied by loss of the wildtype allele in tumor DNA, consistent with a classic tumor suppressor gene mode of inactivation (27). *DIS3* was found recurrently mutated in this cohort, consistent with a recent study that described *DIS3* as a predisposing gene mutated in renal MALT (28, 29). Although described as a tumor suppressor in various cancers, *FAT1* was few found mutated in lymphoma entities to date (30–32). Thus, we first identified potential somatic oncogenic activating mutations of *FAT1* in OAML. Finally, although the mutations in *TTN* and *MUC16* appeared to be relatively common (24% and 10%), these two genes have rarely been recognized as tumor-associated genes (33, 34).

### Distribution and Characteristics of Mutations in *TNFAIP3* and *IGLL5*

**Figure 3:**
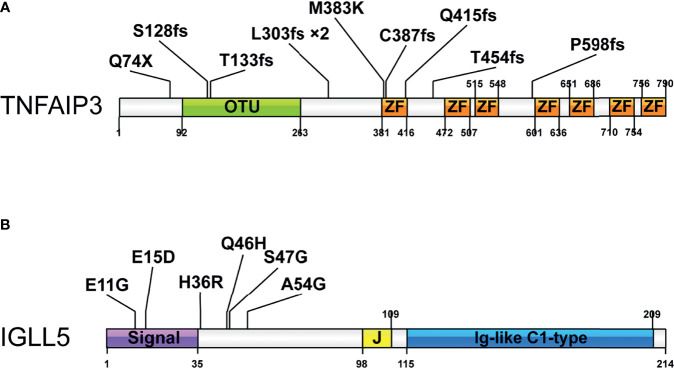
Pattern and distribution of mutations in selected genes. **(A)** Tumor necrosis factor alpha-induced protein 3 (*TNFAIP3*) and **(B)** Immunoglobulin lambda-like polypeptide 5 (*IGLL5*). Colors describe the types of mutations: orange, missense mutation; blue, nonsense mutation; purple, frameshift insertion, or deletion. OTU-Ovarian tumor domain belong to the family of deubiquitinating cysteine proteases; ZF-zinc finger domain; Signal-signal regions; Ig-like C1-type immunoglobulin-like constant 1-type. *2 indicates the mutation occurred in 2 cases.

**Figure 3** shows amino acid changes caused by nonsynonymous mutations in the two recurrently mutated genes. The *TNFAIP3* mutations included frameshift indels (n=8), missense (n=1), and nonsense (n=1) mutations (**Figure 3A**). Most alterations would result in truncation of TNFAIP3, a negative regulator of the NF-κB pathway. The remaining missense change, M383K, was located at the zinc finger domain, which is likely critical for activation of the E3 ubiquitin ligase. These deleterious somatic mutations are indicative of a tumor suppressor role for this gene.

We detected six mutations of *IGLL5*, one of the most notable novel mutation targets discovered in this study. Although *IGLL5* was reported to be recurrently mutated in chronic lymphocytic leukemia (CLL) and diffuse large B-cell lymphoma (DLBCL), none of these SNV were overlapped. All mutations from this study were single nucleotide polymorphisms (SNPs), which were predicted to result in amino acid changes of protein sequence (**Figure 3B**). Most of these mutations are clustered in the N-terminal signal regions, probably resulting in reduced transcript levels according to the prior report (35). These SNVs were less well conserved predicted by Varsome, while analysis using Vector NTI. Advance version 11.0 software showed that several positions (e.g., p.S47) were relatively conserved, supporting that these variants might cause functional changes (**Supplementary Figure 1**). Moreover, three of these variants (p.E11G, p.E15D, and p.Q46H) were described in the Catalogue Of Somatic Mutations In Cancer (COSMIC) database. Together, we thought that these mutations might be likely pathogenic, though available evidence is not sufficient.

### Correlations Between *IGLL5* Alternations and Clinic Characteristics in OAML

Given that *IGLL5* was the most mutant novel gene in our cohort, we compared the clinicopathologic features of patients with and without *IGLL5* mutations, according to a published paper (**Table 2**) (36). Remarkably, in five mutated cases, three patients relapsed in observation, a higher proportion than in the nonmutant group (60% *vs*. 6%, *P*=0.032). Although not statistically significant, patients with *IGLL5* mutations had a higher propensity at advanced stages than those without mutations when diagnosed (80% *vs*. 12%, *P*=0.09). Similarly, a higher proliferation rate (Ki-67 index >10%) seemed to be associated with the *IGLL5*-mutant group (40% in mutant cases *vs*. 6% in nonmutant cases, *P*=0.08). Other clinicopathologic features, such as age, sex distribution, immunophenotype, plasmacytic differentiation and IgH gene rearrangement showed no statistically significant differences between the two groups. In addition, we also detected the global scenario of associations between the *TNFAIP3* mutations and histopathologic features, but no significant correlation was found (data not shown).

**Table 2:** Comparisons of clinical course according to OAML patients with or without *IGLL5* mutation.

Characteristics	mutant *IGLL5* n=5 (24%)	wildtype *IGLL5* n=16 (76%)	*P* value
**Age at diagnosis (years)**			0.525
<55	1/5 (20%)	6/16 (37.5%)	
55-65	2/5 (40%)	8/16 (50%)	
>65	2/5 (40%)	2/16 (12.5%)	
**Sex (Male: Female)**	3:2	9:7	0.647
**Ann-Arbor Stage at diagnosis**			0.090
I	2/5 (40%)	14/16 (88%)	
II	2/5 (40%)	1/16 (6%)	
IV	1/5 (20%)	1/16 (6%)	
**Localization at diagnosis**			1.000
Orbita	4/5 (80%)	11/16 (69%)	
Conjunctiva	1/5 (20%)	3/16 (19%)	
Lacrimal gland	0/5 (0%)	2/16 (12%)	
**Laterality (Right: Left)**	3:2	9:7	0.647
**Treatment after surgery**			0.275
Radiotherapy	2/5 (40%)	5/16 (31%)	
Immunochemotherapy	0/5 (0%)	4/16 (25%)	
Radiotherapy + Immunochemotherapy	2/5 (40%)	1/16 (5%)	
Watch and wait	1/5 (20%)	6/16 (29%)	
**Clinical outcome**			0.032
Remission	2/5 (40%)	14/16 (88%)	
Relapse	3/5 (60%)	1/16 (6%)	
**Proliferation index Ki-67, (%)**			0.080
<10%	1/5 (20%)	11/16 (94%)	
10%-30%	4/5 (80%)	5/16 (31%)	
**Immunophenotype**			0.293
CD5+	1/5 (20%)	4/16 (25%)	
CD10+	1/5 (20%)	0/16 (0%)	
CD5-/CD10-	3/5 (60%)	12/16 (75%)	
**Plasmacytic differentiation**	3/4 (75%)	5/9 (56%)	0.490
Ig κ+/Ig λ-	2/3 (66%)	2/5 (40%)	
Ig λ+/Ig κ-	1/3 (33%)	3/5 (60%)	
**FISH IgH gene rearrangement**	3/4 (75%)	6/12 (50%)	0.392

**Figure 4:**
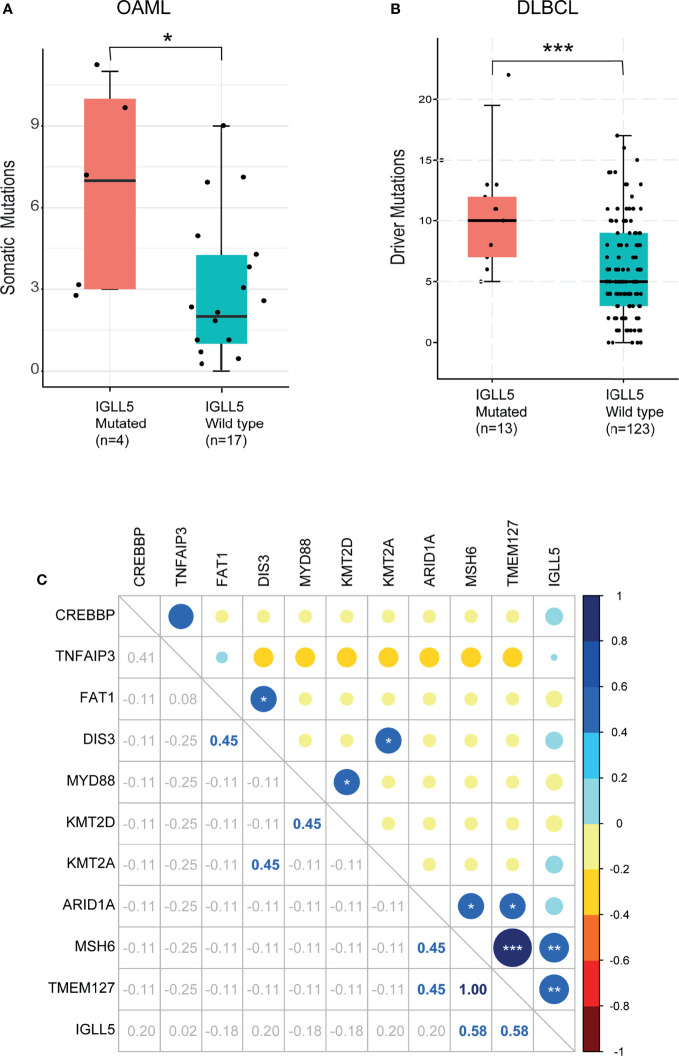
Association of mutant *IGLL5* with clinical characteristics. Box plots of comparison of mutation load between tumors with and without mutations in *IGLL5*. *IGLL5*-mutated tumors presented **(A)** a significantly higher median of somatic driver mutations (3 in mutant cases *vs*. 7 in wildtype cases) in this study and **(B)** a significantly higher median of driver mutations (5 in mutant cases *vs*. 10 in wildtype cases) in data from the TCGA online database. **(C)** Heatmap shows co-occurrence and mutual exclusivity identified by WES across mutation types. Numbers indicate Correlation coefficients analyzed by the Pearson product-moment correlation coefficient. *P* values were calculated using the Mann-Whitney U-test. TCGA pan-cancer data were obtained from the cBioPortal (https://www.cbioportal.org). (*P < 0.05, **P < 0.01, ***P < 0.001).

Moreover, *IGLL5* was associated with higher mutation loads, with seven somatic mutations/cases compared to three wildtype cases (*P*=0.04, **Figure 4A**). A similar result was obtained in DLBCL with public datasets from The Cancer Genome Atlas (TCGA), with 10 in *IGLL5* mutated cases *vs*. five in the wild types (p<0.01, **Figure 4B**). Together, these observations indicate that cases with *IGLL5* variants might harbor intrinsic defects resulting in an unstable genome (37). To gain further insight, **Figure 4C** reveals the significant mutation co-occurrence within the top 11 somatic mutations (>10%) across the cohort. Although the analysis of co-occurrence suffers from weak statistical power, we detected significant associations between pairs of genes: 1) *IGLL5* and *MSH6* (R=0.58, *P*<0.01) as well as *TMEM127* (R=0.58, *P*<0.01) as expected; 2) *TMEM127* and *MSH6* (R=1, *P*<0.001) as well as *ARID1A* (R=0.45, *P*<0.05), consistent with their anti-carcinogenic roles in different tumors (26, 27, 38). Together, these analyses support the hypothesis that *IGLL5* might combine activation of multiple tumor suppressor genes that may be needed to drive lymphomatosis in OAML.

### Distinct Somatic Mutation Spectrum of OAML in Asia

**Figure 5:**
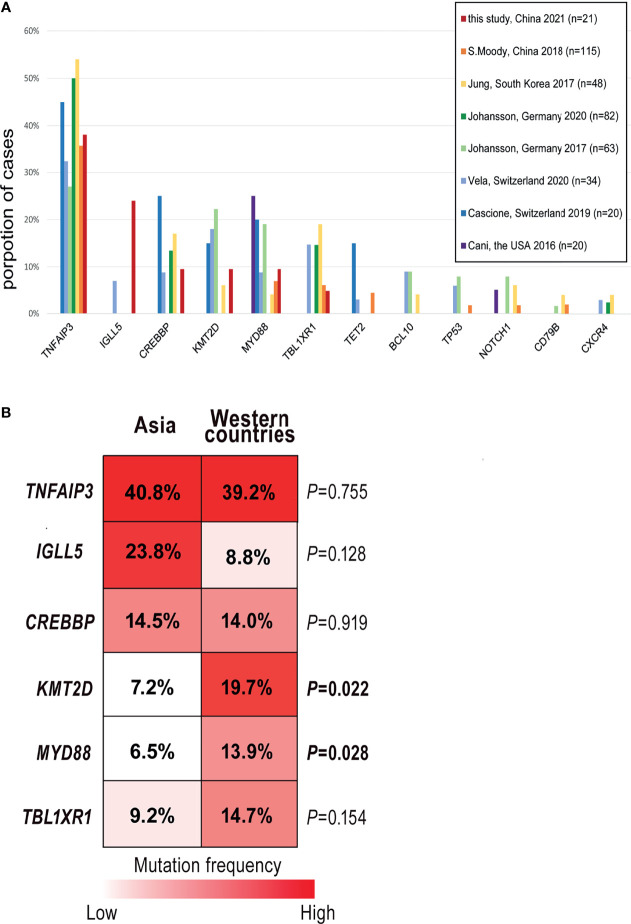
Comparisons of the mutation frequencies from different studies. **(A)** Column graphs show the proportion of 11 common mutated genes reported by seven previous studies in ocular extranodal marginal zone lymphoma of mucosa-associated lymphoid tissue (OAML). Each column represents a gene ranked in decreasing order of average detection frequency in the tumor. **(B)** Stacked column plots display the comparisons of the five most common genes detected in this study. Mutation rates were calculated by dividing the number of cases carried mutant gene by the study cohort’s size and total the number of cases with mutations from 8 studies.

So far, seven pieces of research have described the mutational patterns of OAML based on NGS (12, 15–17, 22, 39, 40). To define whether the somatic mutation signatures of ocular MALT lymphoma are distinct or overlapped in different regions, we analyzed all these studies (n=382) with a total of 168 genes discovered by NGS in OAML and compared their findings with our results (**Supplemental Table 3**). Finally, 11 frequently-occurring genes were selected, as shown in **Figure 5A**. The most frequently mutated genes as reported previously were all detected in the present study, including *TNFAIP3* (38% in this study *vs*. an average 39%, range 27-45%, from previous studies), *CREBBP* (10% *vs*. 16%, range 3-25%), *MYD88* (10% *vs*. 14%, range 4-25%), *KMT2D* (10% *vs*. 15%, range 5-22%), *TBL1XR1* (5% *vs*. 14%, range 5-19%). Genes with low mutation rates (<10%) such as *TET2, BCL10, TP53, NOTCH1*, *CD79B*, and *CXCR4* were not detected in the present study. This may be due to the lower sensitivity of WES compared to TGS, as well as sample heterogeneity.

Although these OAML studies broadly shared a typical mutation signature characterized by variants of *TNFAIP3*, *CREBBP*, and others, the somatic mutation frequencies differed between Asian and Western OAML cohorts, especially those from Europe (15, 22, 39), as shown by **Figure 5B**. For *KMT2D*, a critical molecule involved in chromatin remodeling/transcription regulation, the percentages of mutated cases were lower in Asia than in other regions (7.2% *vs*. 19.7%, *P*=0.022). Similarly, the mutation frequency of *MYD88* was relatively low in Asia (6.5% *vs*. 13.9%, *P*=0.028). The same mutation abundance was observed in *TBL1XR1*, an essential factor in transcriptional repression mediated by unliganded nuclear receptors, although the analysis suffered from a lack of statistical power.

### Novel Pathways Involved in OAML

**Figure 6:**
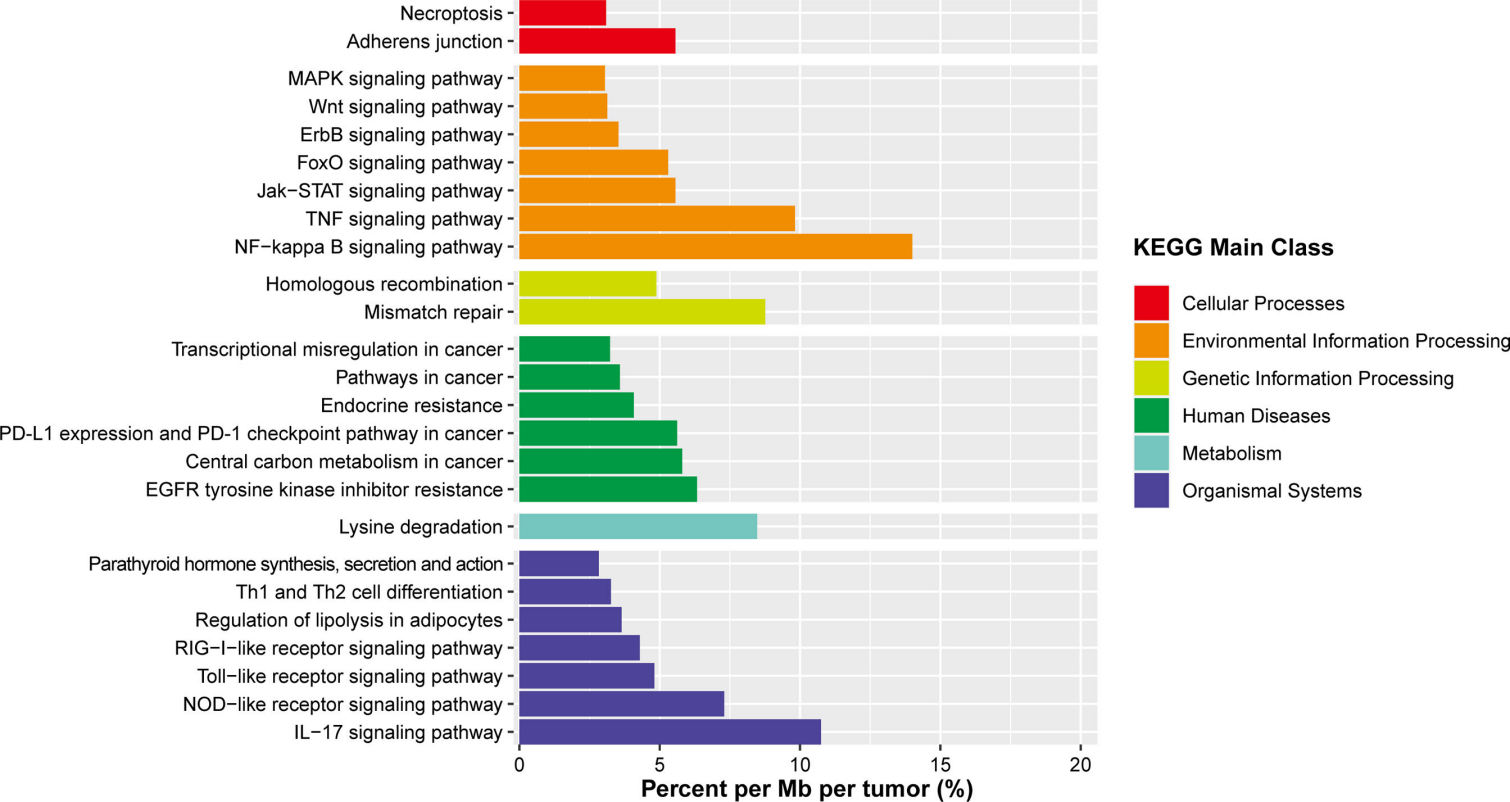
Summary of signaling pathways affected by frequent somatic mutations in OAML. In addition to the common mutations reported, several novel mutations are described, affecting defined biological pathways. Mutation rates were calculated by dividing the total number of mutations encountered in a pathway by the number of all genes belonging to that pathway and the size of the coding tumors genome.

In the whole-exome sequencing cohort, somatic mutations were most common in BCR/NFκB, chromatin-modifying, and immune surveillance pathways. However, a considerable proportion of novel, previously unrecognized targets of somatic alterations detected in this study suggested additional/distinct tumorigenic pathways in OAML. Thus, we further explored the canonical signaling pathway using KEGG pathway analysis (**Figure 6** and **Supplemental Table 4**). The results showed that these targets were directly involved in the pathways correlated with signal transduction (NF-κB, TNF, JAK-STAT signaling pathways) and the immune system (IL-17, NOD-like receptor signaling pathway). In partial agreement with recent research, the Jak-STAT signaling pathway was also targeted in several OAML tumors (5.56%) (22).

Notably, the mismatch repair (MMR) pathway was never previously recognized in OAML. However, it was reported mutated at a comparatively high rate (8.7%) in the present study, mainly due to mutations of *MSH6* detected in two tumor samples. In light of the fact that the MMR deficiency may contribute to a higher mutation burden, we compared cases with and without *MSH6* mutations. These tumors presented a median number of mutations much higher than the remaining OAML cases (10.5 *vs*. 3, *P*=0.01), but not in frameshift mutations (*P*=0.467). Such an analysis would be inconclusive given the small sample size of subgroup, but it is still partially confirmed with the previous finding in the Chinese DLBCL cohort (41).

## Discussion

Recently, comprehensive characterization of several cancer genomes has been made feasible due to the rapid advances in NGS technologies. The high prevalence of mutations discovered in the BCR/NF-κB signaling components (including *TNFAIP3* and *MYD88*) was one of the most significant findings from NGS studies and highlighted the constitutive activation of the BCR/NF-κB signaling pathway in the progression of OAML (4). However, these somatic mutations were identified in a minority of OAML cases, and reports from different NGS studies have shown significant differences in the mutation frequencies for particular genes. These problems may be due to intratumor heterogeneity (different variants with low allelic fractions between FF and FFPE tissue specimens), genomic heterogeneity among different populations, and biased sequencing strategies (high throughput sequencing based on the reported genetic lesions). We aimed to minimize such flaws by performing unbiased WES on 21 cases to characterize the landscape of mutated genes and identify more potential drivers in OAML.

Our study observed mutations in previously known OAML targets such as *TNFAIP3*, *MYD88*, *CREBBP*, *KMT2D*, and *TBL1XR1* but also identified novel or previously unrecognized mutation targets in OAML such as *IGLL5*, *MSH6*, *DIS3*, *FAT1*, and *TMEM127*. However, genes such as *MYD88* and *KMT2D* were mutated at lower frequencies than previously reported, and some already identified genes were not detected. *IGLL5* was the most notable novel mutation target in the current cohort, affected by nonsilent mutations in 24% of tumors. In general, little is known about the function of *IGLL5*, but it encodes a surrogate light chain that plays a critical role in B-cell development. In normal physiological conditions, its expression activates B lymphocyte differentiation and the memory immune response, and it is frequently mutated in human B cell malignancies, including chronic lymphocytic leukemia (35), diffuse large B-cell lymphoma (41), and Burkitt lymphoma (42). Of note, while *IGLL5* has been described as one of the most commonly mutated targets in these series, the clinical relevance of these mutations is controversial. Several studies identified mutated *IGLL5* with prognostic value (35, 43, 44), whereas others found no association between mutated genes and patients’ outcomes (45). Our data suggested that mutant *IGLL5* might be linked to an early recurrence. However, histopathologic features are similar in patients with and without *IGLL5* mutation, including immunophenotype, plasmacytic differentiation and IgH gene rearrangement. The significate of the proliferation index is not statistically precise given the small samples. Further studies involving a more extensive series of patients are warranted to shed more light on the potential clinical and pathologic correlations in OAML.

Currently, the pathogenic mechanism of mutant *IGLL5* remains to be determined. A recent study has suggested that mutated *IGLL5* showed a trend towards reduced transcription compared with wild type in CLL (35). Based on the oncomine database (https://www.oncomine.org), although no differential expression of *IGLL5* mRNA was found, its homologous genes (*IGLL1*, *IGLL2*, and *IGLL3*) showed a lower expression in lymphoma (*P <*0.05, Fold Change>2, **Supplementary Figure 2**). The TCGA database showed that the transcript level of *IGLL5* was significantly below normal in several tumors, especially in DLBCL (**Supplementary Figure 3**). However, Kaplan-Meier survival analysis based on the TCGA indicated that patients with lower *IGLL5* mRNA levels had shorter overall survival than patients with higher levels (*P* =0.048, **Supplementary Figure 4**). In view of this, we speculate that *IGLL5* mutations may be associated with an increased risk of OAML pathogenesis due to reduced mRNA expression. However, mutant *IGLL5* may suggest off-target activation-induced cytidine deaminase (AID) activity (35). AID was described to resolve genetic lesions caused by the error-prone DNA polymerase η(eta) during B-cell development, which might explain the somatic hypermutation in the cases with mutated *IGLL5* (46, 47). Although their functional significance remains unknown without further experimental work, these results at least indicated that the dysregulated AID. activity might be involved in the formation of OAML. Our results suggest that patients with mutated *IGLL5* harbor significantly more mutations and may support this hypothesis. In conclusion, despite the pathogenic mechanism being poorly defined, frequent mutation of *IGLL5* in OAML through NGS diagnostics could emerge as a novel risk marker.

*TNFAIP3* was another frequently mutated gene in the present study, in line with previous reports (40). This gene coding for A20, a negative global regulator of the canonical NF-κB pathway, is recurrent in OAML. The majority of *TNFAIP3* mutations were deleterious changes, leading to increased NF-κB and p38 activity. The observation of A20 defects in this cohort may suggest a possible therapeutic approach *via* inactivation of the NF-κB pathway. However, provided that the NF-κB pathway regulates a broad spectrum of physiologic functions, including the development of the immune system, it is important to consider that inactivation could simultaneously result in deleterious side effects.

Since the novel mutation targets discovered by this study, especially those with a higher frequency (>10%), may play a notable role in lymphomagenesis, we performed pathways analysis. Apart from the report that the driver genes mainly coded for proteins involved in the BCR/NF-κB pathway, our results showed that alterations also occurred frequently in genes affecting the additional pathway, including MMR (e.g., *MSH6*), TNF, and IL-17 signaling pathways (e.g., *IKBKB*, *TRAF3*). As the present cohort is small, the frequencies of cases with pathogenic somatic alterations in this study are likely an overestimate of the frequencies in the population of OAML patients. A future follow-up study is required to explore outside the scope of this paper.

The MMR machinery is a critical pathway for maintaining genome stability by recognizing and directing repair of base-base mismatches and insertion/deletion after DNA damage. Microsatellite instability (MSI) is a consequence of DNA mismatch repair (MMR) deficiency, which refers to the hypermutator phenotype secondary to frequent polymorphism in short repetitive DNA sequences and single nucleotide substitution (26, 48, 49). Similar to previous studies (41, 50), our study demonstrated a higher mutation burden in cases underlying mismatch repair defects, besides frameshift mutations. The null finding of such analysis would be inconclusive given the small sample size and lack statistical power. In addition, the neoantigens caused by high frequencies of nonsynonymous mutations can trigger a more robust and long-lasting immune response and strong tumor-infiltrating lymphocytes (TIL) infiltration with tumor eradication (51). Preclinical studies also supported the idea that tumors showing microsatellite instability-high or a high number of TIL phenotype will be more responsive to checkpoint immunotherapies (52–54). Our discovery that MMR mutated in a proportion of OAML cases may support the development of therapeutic strategies in these patients. Regrettably, due to orbital biopsy specimens were small, the remaining samples were inadequate for further molecular analysis. It is well beyond the current data to explore the new frontiers of MMR genes in OAML, but this is an exceedingly important direction for future efforts as we begin large-scale dissemination efforts.

Additionally, as the first analysis of the OAML coding genome in a Chinese cohort, this study shows distinct somatic mutation profiles between OAML cohorts from East Asian and Western countries. Given that distinct somatic mutation patterns based on geographical variations were also described in DLBCL (41, 55), such differences cannot be explained solely by sample size bias. In particular, the mutation frequencies of overlapped genes were not significantly different from those of a prior Chinese cohort of 115 OAML cases (40). Since the germline genetic variants have been shown to promote the selection and generation of specific somatic mutations during tumorigenesis, these distinctions might be caused by the different genetic backgrounds of Asian and Western populations (34, 56). Moreover, exploration of specific etiological agents such as infectious disease or dietary habits could result in different oncogenic pathways. Indeed, cohorts from some regions (Italy, South Korea, Germany, and Austria) present with chronic *Chlamydia* spp. infection at diagnosis, whereas those in other regions (Japanese and Danish) do not (6–9, 11, 13). These findings further imply that characterization varies between populations (9).

Finally, several limitations should be noteworthy. 1) As orbital biopsy specimens are often small, the specimen sections usually do not show typical immunophenotype of MALT. In addition, FISH assay failed to detect clonal IgH gene rearrangement in 7 cases of OAML in our cohort, which might be due to the sensitivity of the FISH assay alone may not be adequately high to detect all clonal issues. 2) The bias may exist due to the small sample size, especially for specific subgroups. Moreover, due to the consumption in the previous experiment, the remaining specimens were insufficient for further pathological analysis. The results must be interpreted with caution. 3) It is essential to note that different technical platforms and analysis pipelines may lead to variable results. Previously published NGS studies, as well as our own, have limited power to detect those cancer driver genes that are mutated in <5% to 10% of samples. Inter-tumor heterogeneity carries essential implications for OAML patients’ management, as targeted therapies aim to explore specific genetic alterations in tumors. Our findings highlight the need to characterize a more significant number of OAMLs derived from different populations and refine the mutation spectrum based on the molecular defects discovered by the NGS studies.

## Conclusions

In summary, our findings identified a novel gene, *IGLL5*, recurrently mutated in 24% of OAML tumors and associated with a higher recurrence rate. Our results compared with those of previous studies highlight the genetic heterogeneity of OAML between Asian and Western individuals, which should be further validated in larger cohort populations in the future.

## Data Availability Statement

The datasets presented in this study can be found in online repositories. The names of the repository/repositories and accession number(s) can be found in the article/**Supplementary Material**.

## Author Contributions

Conceptualization: HL, WX, and JL. Methodology: FW. Software: AZ. Validation: YW, FW, and AZ. Formal analysis: AZ. Investigation: AZ. Resources: FW. Data curation: YW. Writing—original draft preparation: AZ. Writing—review and editing: FW and YW. Visualization: AZ. Supervision: WX. Project administration: HL. Funding acquisition: JL. All authors contributed to the article and approved the submitted version.

## Funding

This research was funded by the National Natural Science Foundation of China, grant number #81720108002, and the National Natural Science Foundation of China (No. 82100206).

## Conflict of Interest

The authors declare that the research was conducted in the absence of any commercial or financial relationships that could be construed as a potential conflict of interest.

## Publisher’s Note

All claims expressed in this article are solely those of the authors and do not necessarily represent those of their affiliated organizations, or those of the publisher, the editors and the reviewers. Any product that may be evaluated in this article, or claim that may be made by its manufacturer, is not guaranteed or endorsed by the publisher.
