# Supplementary material for: Analysis of Genetic Alterations in Ocular Adnexal Mucosa-Associated Lymphoid Tissue Lymphoma With Whole-Exome Sequencing
Source: Front Oncol. 2022 Mar 11;12:817635. doi: 10.3389/fonc.2022.817635 (PMC8962736; doi:10.3389/fonc.2022.817635)
Supplement: Supplementary file 5 [file DataSheet_1.docx]

Supplementary Material

# Supplementary Data

Supplementary Material was uploaded separately on submission.

# Supplementary Figures and Tables

##
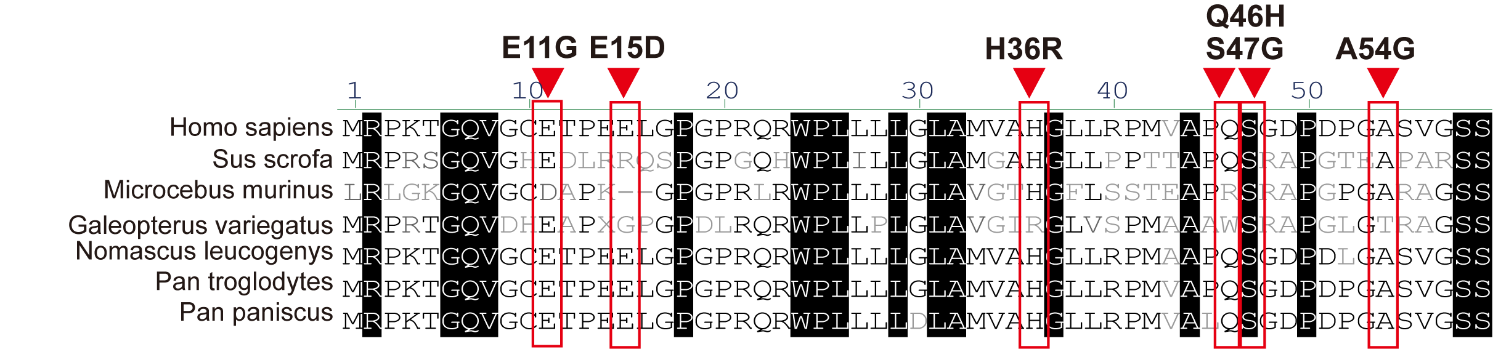
Supplementary Figures

**Supplementary Figure 1.** Multispecies alignment of the N-terminal portion of IGLL5, encoded by the first exon of the gene, was performed by Vector NTI Advance11.0 software.


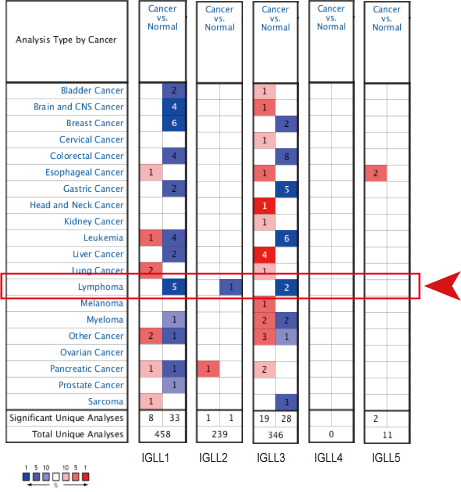


**Supplementary Figure 2.** Comparing the mRNA level of IGLL5 with its homologous genes in tumor and normal tissues of different cancer types in Oncomine database(https://www.oncomine.org/).


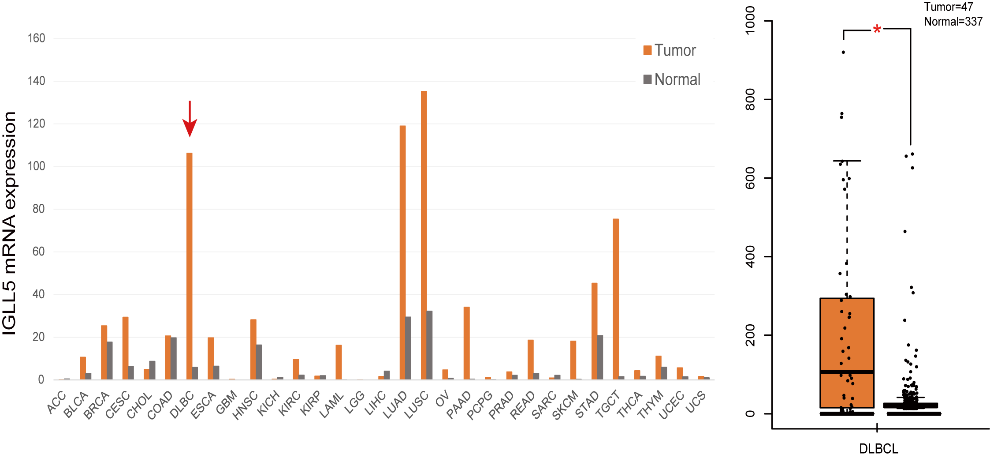


**Supplementary Figure 3.** Comparing mRNA expression levels of IGLL5 in tumors and adjacent normal tissues of various cancer types based on TCGA database (left), and the level of IGLL5 mRNA was significantly decreased from normal lymphatic tissue to diffuse large B-cell lymphoma (right).


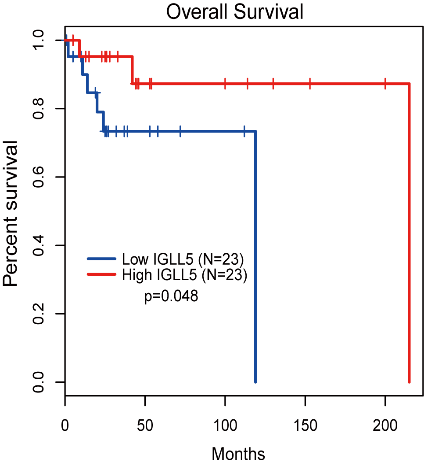


**Supplementary Figure 4.** Kaplan–Meier survival analysis curve calculated from 47 DLBC patients from TCGA database.
